#  Knowledge of warning signs, presenting symptoms and risk factors of coronary heart disease among the population of Dubai and Northern Emirates in UAE: a cross-sectional study 

**DOI:** 10.3126/nje.v7i2.17973

**Published:** 2017-06-30

**Authors:** Nelofar Sami Khan, Syed Ilyas Shehnaz, Gomathi Kadayam Guruswami, Salwa Abdelzaher Mabrouk Ibrahim, Sana Abdul Jabbar Mustafa

**Affiliations:** 1 Associate Professor, Dept. of Biomedical Sciences, Gulf Medical University P.O.Box 4184, Ajman, UAE.; 2 Department of Pharmacology, Faculty of medicine, Annamalai University Chidambaram, India; 3 Professor and Head, Department of Biomedical Sciences, Gulf Medical University Ajman, UAE; 4 Consultant & adjunct associate professor of internal medicine & deputy medical Director, Thumbay Dubai hospital, Dubai UAE; 5 Lab Technician, Department of Biomedical Sciences, Gulf Medical University Ajman

**Keywords:** Coronary heart disease, heart attack, United Arab Emirates, Young adult population, Middle Easterners

## Abstract

**Background:**

To evaluate the level of knowledge regarding warning signs, presenting symptoms and risk factors associated with coronary heart disease (CHD) among population of Dubai and Northern Emirates in UAE.

**Materials and Methods:**

A cross sectional survey of 1367 residents of Dubai and Northern Emirates was conducted using a self-administered questionnaire.

**Results:**

Respondents were classified into two groups: Young Adult Population (YAP; 18-24 years of age) and General Population (GP; 25 years and older). Majority of participants were males (56.7%) and of South Asian (57.5%) or Middle-Eastern (30.8%) ethnicity. Regarding presenting symptoms of CHD, chest pain was identified by around 80% of population, whereas pain in the left shoulder was recognized by 61% of GP and 44% of YAP. Atypical symptoms were poorly identified. Regarding risk factors, only one-fourth population knew that males were at higher risk compared to premenopausal females. Few knew that the risk increases in females after menopause and that the risk is higher for females who smoke and use oral contraceptives. 62% knew that the survivors of a heart attack are at high risk of recurrences. Except for tobacco smoke, hypercholesterolemia and hypertension, knowledge of other risk factors was not satisfactory. Older adults and females had comparatively higher level of knowledge.

**Conclusion:**

Knowledge level of many of the symptoms and risk factors of CHD is unsatisfactory. There is, therefore, a need to increase the awareness in the population of UAE. The knowledge gaps identified through this study can be addressed through health campaigns to increase the awareness about warning signs, symptoms and modifiable risk factors. .

## Introduction

Coronary heart disease (CHD) has been reported as the number one cause of death in many countries, with certain ethnic groups being more susceptible to early onset of serious problems, resulting in higher premature deaths [ 1 ]. Lifestyle factors and genetics were the major contributors to the high morbidity and mortality associated with it [ 2 ]. Since lifestyle risk factors are modifiable, public can reduce their risk of heart disease by increasing their awareness, and simultaneously adopting healthy behaviors in routine life [ 3, 4 ]. Timely identification of warnings and symptoms of CHD facilitates prompt presentation to the hospital for immediate treatment as time susceptible interventions reduces mortality and morbidity [ 5 ]. Hence, the most important contributor for better outcome rests on the patient ' s ability to recognize the early indicators of CHD [ 6 ]. 

 United Arab Emirates (UAE) has gone through rapid economic progression in the last four decades and ranks second in the Gulf Cooperation Council (GCC: a union of Bahrain, Kuwait, Oman, Qatar, Saudi Arabia, and the UAE) in terms of economy [ 7 ]. Its population is majorly urban (85%) and multiracial with the following ethnic groups: Emiratis (UAE nationals;19%), other Arabs & Iranians (23%), South Asians (50%), and other expatriates like Westerners, Africans & East Asians (8%) [ 8 ]. 

 Non-communicable diseases account for 65% of total deaths in UAE, with 30% due to cardiovascular diseases (CVD) [ 9 ]. Moreover, the morbidity data reports high incidence of CVD risk factors like diabetes and hypertension. There is, therefore, a need for health promotion programs. For the successful development and implementation of such programs, it is essential to evaluate the baseline knowledge of the target population [ 10 ]. Based on the outcome, a health educational program appropriate to this multicultural society can be designed or an existing program modified to fit to this community. To the best of our knowledge, no study has been conducted in UAE to assess the community understanding of symptoms and risk factors of CHD. 

 The main objective of this study was to evaluate the level of knowledge regarding the warning signs, presenting symptoms and risk factors associated with CHD among the population of Dubai and Northern Emirates in UAE. Secondary objective was to determine the association between age, gender, ethnicity and economic status and the level of knowledge. Ultimately, it will identify the knowledge gaps and help in developing the programs aiming to reduce the burden of this disorder in the UAE society.

## Methodology

### Study design and the participants:

A cross-sectional questionnaire- based survey was conducted among a convenience population sample from Dubai and Northern Emirates in UAE during January 2014 to December 2015. The participants were broadly classified into two groups: Young Adult Population (YAP; 18 to < 25 years of age) and General population (GP; 25-60 years). The population was further subdivided into three groups based on ethnicity: Middle Easterners (ME) - including Emiratis, other Arabs & Iranians; South Asians (SA) – mainly Indians, Pakistanis & Bangladeshis; and the rest grouped as “others” which included Europeans, Americans, Africans & South-East Asians.

### Sample size calculation:

For the calculation of sample size, it was assumed that the 50% (p=0.5) of the population has the knowledge regarding the risk factors of Cardio Vascular Disease and the statistical significance as 5%. The margin of error was taken as 10% of the prevalence (L=0.05). Thus, the minimum required sample size was calculated as 400; 400 males and 400 females were selected separately. The total samples included in this study is 1367 which is more than the minimum required sample.

### Data collection and inclusion criteria:

A research assistant was recruited for data collection. The GP in the market places, parks, offices, religious gatherings and clinics were contacted. Voluntary informed consent was obtained from all respondents. The questionnaire was self-administered by the literate participants in English or Arabic language. For less educated individuals, the research assistant was trained to translate the information into two Indian languages namely Hindi and Malayalam; and a Pakistani language (Urdu).

For the YAP group, students in various universities and colleges were contacted and the questionnaire was self-administered after explaining the objectives of the study. A convenience sample of 18 educational institutions were chosen across Dubai and Northern Emirates in UAE in order to attain diversity of the YAP.

### Exclusion criteria:

Healthcare related professionals and healthcare related students were excluded from the study. Individuals who didn’t understand English, Arabic, Urdu, Hindi or Malayalam were also excluded.

### Questionnaire design and validation:

The questionnaire was developed after extensive literature search [ 3, 11, 12 ]. Throughout the questionnaire the word " heart attack " was used for the understanding of the general public. It was divided into several sections:

Section A elaborated the demographic and socio-economic information of the participants; 

 Section B expounded the definition of CHD/heart attack and respondents were asked to tick as " I knew this " or " I didn 't know this " ; 

 Section C detailed the early warning signs (indicating high susceptibility of getting heart attack) and the presenting symptoms of heart attack. Participants were asked to select " yes "," no " or " unsure " for each option; 

 Section D enlisted various factors which increased the risk of getting heart attack and the respondents had to indicate as " yes " , " no " or " unsure " for each option. 

 Section E included the following statements which were to be answered by ticking " I know this " or " I didn ' t know this ":

1. Risk of CHD is high in women who smoke and use oral contraceptives;
2. The risk of CHD increases in women after menopause;
3. Survivors of heart attack are at high risk of recurrences and at high risk of dying from it.

The questionnaire was initially developed in English language. Content validity was done by experts in the field of internal medicine and cardiology. It was then pilot tested on ten persons of each of the YAP and GP groups. Modifications were made based on their suggestions to increase the clarity of each statement. 

 The questionnaire was translated into Arabic language by a native Arabic speaker followed by back translation into English. The Arabic questionnaire was also pilot tested in order to remove inconsistencies.

### Ethical committee approval:

The study got approval by the Gulf Medical University Institutional review board. The research was conducted in accordance to 64th WMA, general assembly, Fortaleza, Brazil, October 2013, Helsinki - ethical principles for medical research involving human subject's guidelines.

### Outcome Variable:

The primary outcome variable was the knowledge level regarding the warning signs, presenting symptoms and risk factors associated with CHD.

### Explanatory variable:

Age, gender, ethnicity and economic status were the explanatory variables.

### Data management and statistical Analysis:

Data was entered on excel spread sheet and analyzed using SPSS version 23. The Cronbach’s alpha for the questionnaire was 0.8. For calculating “overall” level of knowledge about signs & symptoms or risk factors, one point was giving to each correct option and zero for the incorrect option. A maximum of 13 points were given to the knowledge of signs and symptoms and 15 to the risk factors. Knowledge was considered satisfactory if the overall score for the section was more than 50% of maximum score. This arbitrary standardization was done to compare the knowledge level among various groups.

From the tabulated data, overall percentages of correctly identified options for each sign & symptom were calculated. Comparative statistics was carried out based on: age, gender, economic status and ethnicity. Age was recoded from a continuous variable to a categorical one. Chi-square test of significance was applied for comparisons. A p value of < 0.05 was regarded as statistically significant.

## Results

Out of 1600 people contacted, around the 1367 adult UAE residents responded (response rate: 85%). The majority of the sample was males, educated, belonging to multi-ethnic groups and higher socio-economic strata. The socio-demographic details of the sample are elaborated in Table 1

Table 2 compares the knowledge regarding definition, warning signs and presenting symptoms of heart attack. The GP were found to have significantly higher level of knowledge. Females of both the groups also possessed significantly higher knowledge scores as compared to the males. No significant difference were observed in overall knowledge scores based on ethnicity. However, in the GP group, SA had significantly more knowledge of pain or discomfort in jaw, shoulder and chest as a symptom of heart attack. ME also recognized breaking out in cold sweat and feeling light headed or faint significantly more than the other groups.

Table 3 assesses the knowledge of risk factors among the GP and YAP. The GP were significantly more knowledgeable than the YAP. Moreover, the females of the GP group had significantly higher knowledge of risk factors compared to males. Though there were no gender differences in the overall knowledge scores in the YAP group, males were more aware of tobacco smoking being a risk factor and females were more knowledgeable about unhealthy diet. There were no differences in overall knowledge scores based on ethnicity. In both the GP and YAP groups, SA identified stress as a risk factor significantly more than other ethnic groups. In the GP group, ME were more aware of increased risk as the age advances generally and specifically for women after menopause. No significant differences in knowledge were observed based on economic status and educational level (Not shown in tables).

## Discussion

As an initial step in assessing health education needs of the population, we have evaluated the knowledge related to CHD among the population in UAE. Timely identification of warnings and symptoms of CHD by the patient and family members facilitates prompt presentation to the hospital. Moreover, the most important contributor for better outcome rests on the ability to recognize the early indicators of CHD.

### Knowledge of warning signs and presenting symptoms of CHD

Our results revealed that half of the total population scored a satisfactory benchmark in the overall understanding of signs and symptoms, with significantly higher number of GP compared to YAP. Females in our study sample were observed to be more knowledgeable than males, as also reported in other studies [ 11, 13 ]. Though disparity in the knowledge level based on socioeconomic status was also documented elsewhere [ 12, 14 ] our population didn't show such difference, probably due to less participation from lower socioeconomic groups. 

 The common man generally considers an acute CHD patient to be someone suffering sudden stabbing pain in the chest, grabbing his chest, and collapsing while atypical symptoms like shoulder pain, dyspnea, nausea, or syncope are less acknowledged [ 15 ]. In accordance, majority of our respondents (80%) identified chest pain as presenting symptom while atypical symptoms were less recognized. Literature, however, reveals that one-third of the patients with definite diagnosis of heart attack don't exhibit chest pain [ 16 ] and absence of chest pain or discomfort is a potent predictor for missed diagnosis and delayed treatment [ 17 ]. This accentuates the need to increase awareness about both the typical and atypical signs of CHD in the general public. 

 Though the knowledge of half of the population of UAE was not satisfactory, but weighing against other similar studies, our population in general and the ME (Arabs) in particular have higher knowledge compared to the Kuwaiti natives (locals), regarding chest, shoulder & arm pain; shortness of breath; and feeling lightheaded or faint as a symptom of CHD [ 13 ]. It is also heartening to observe that our population has higher knowledge of CVD signs and symptoms compared to the general population of Pakistan [ 10 ] and Nigeria [ 18 ]. Though the knowledge level reported from the rural/non-rural Americans [ 12 ] and Singaporeans [ 11 ] was very high, the CVD awareness of our sample was more comparable to that of American Indians and Alaska natives [ 19 ]. Analogous to our observations, older respondents and females in Kuwait were found to be more knowledgeable than the other subgroups [ 13 ]. Comparing the knowledge of females of our GP group with the knowledge from a study conducted on secondary level educated females in Malaysia, their understanding level was high for most of the parameters except for shoulder pain [ 20 ]. Similarly, the Hispanic males from US were also found to have higher knowledge compared to the UAE population in general and males in particular [ 21 ]. It should be underscored that less identification of these symptoms increases the risk of not getting timely medical help thus increasing the rate of morbidity and mortality.

### Knowledge of risk factors of CHD

It was observed that around 30% of our respondents had poor awareness about CHD risk factors, as evident from their inability to score the satisfactory benchmark. Our older participants (GP group) and the females of the GP group were significantly more knowledgeable, as also reported in other studies [ 3, 13, 22 ]. However, unlike higher level of education and socioeconomic status being predictors of better level of CHD knowledge in other studies [ 3, 13, 22 ], no difference based on these factors and ethnicity were observed in our sample. Modifiable risk factors are the keystone in the prevention of CHD. If the population, especially the youth, have good knowledge of CHD risk factors, they will be able to embrace primary preventive measures earlier in their lives. 

 It is assuring that smoking was identified as a risk factor of CHD by majority of participants as smoking is responsible for one third of the mortality associated with CHD [ 23 ]. However, only half of them recognized secondhand smoke as a risk factor. Akin to other studies [ 13, 18, 22, 24 ], merely half of our population failed to link diabetes to CHD, although people with diabetes have several folds' higher risk of getting CHD. Further, very few were aware of risk factors like male gender, increasing age, menopause and concurrent use of tobacco and oral contraceptives in females. The GP, females and ME were more knowledgeable of the association between menopause and CHD. Predictably, the GP were also more aware of stress being a risk factor. Females were more knowledgeable about the risk of unhealthy diet and being overweight /obese, probably attributed to their increased concern about health and looks [ 24 ]. 

 Our sample fared better in their knowledge of CHD risk factors as compared to the populations of Pakistan [ 10 ] Nigeria [ 18 ] and Egypt [ 25 ]. Our young respondents, nevertheless, had less knowledge about smoking, high blood pressure and blood cholesterol, diabetes, stress, unhealthy diet, family history, increasing age and male gender compared to Pakistani university students [ 26 ]. Regional concurrence between our sample (the ME group) and Kuwaiti population was observed with almost identical level of knowledge of smoking, diabetes mellitus, stress and family history as risk factors [ 13 ]. Furthermore, in comparison to Malay females, our older females (GP group) were more knowledgeable in few parameters and less in some others [ 20 ]. Similar mixed results were also observed in comparison with studies from USA [ 3, 22 ]. 

 Various UAE government bodies have invested in a number of health promotional campaigns aimed at increasing community awareness, altering the UAE residents' attitudes toward healthy lifestyles and facilitating adoption of healthy practices [ 27 ]. Our results, however, reveal that these public health initiatives have not been entirely successful in targeting all sections of population especially the youth, males and specific ethnic groups. 

## Strengths of the study

The design of the present study, the questionnaire and selection of a convenience sample suits the purpose of our study as it is a preliminary step in assessing health education needs of the community. Demography of many of the sub-groups of our participants reflects the actual demography of the population of UAE.

## Conclusion

Our population-based survey documents the current level of knowledge regarding CHD among the UAE population. Except for chest pain as a presenting symptom and tobacco smoke, hypercholesterolemia and hypertension as modifiable risk factors, which were identified by around 80% of the population, knowledge of rest of the parameters needs to be improved. The data from this study can form the foundation in developing various health promotion programs utilizing available resources. Timely health-promotion and prevention efforts can exert greater impact on quality of life than therapeutic interventions alone commenced at a more advanced age. Modifiable risk factors are the keystone in the prevention of CHD. As evident from our data, it is essential to target the youth and not just the old and middle-aged population. If the population, especially the young people, have good knowledge of CHD risk factors, they will be able to embrace primary preventive measures in their everyday lives from younger ages.

### Limitation of the study:

The limitations of our study are missing segments of population due to language barriers and participation of only literate young population.

### Future scope of the study:

Our results can help to design effective public health campaigns for enhancing the knowledge of risks factors and symptoms of CHD.

### What is already known on this topic?

To the best of our knowledge, our study is the first to be conducted in UAE to assess the community understanding of symptoms and risk factors of CHD.

Results of this study indicate a need to increase awareness regarding the warning signs, presenting symptoms and risk factors of coronary heart disease

Knowledge gaps identified can be used to develop various health promotion programs.

### What this study adds:

As an initial step in assessing health education needs of the population, we have evaluated the knowledge related to Coronary Heart Disease among the population in UAE.

## Figures and Tables

**Table 1:** Demography of the participants

Characteristics	Groups		No. of Participants	Percentage
**Type of Participants Age in years**	Young Adult Population (YAP) 18 to < 25 years (Median age = 20 years)		701	51.3
	General Population (GP) 25 to 60 years (Median age = 34 years)		666	48.7
	**Total**		**1367**	**100**
**Gender**	Male		769	56.7
	Female		588	43.3
	**Total**		**1357**	**100**
	Not reported		10	-
**Ethnicity**	Middle Easterners **(ME)** (N=389; 30.8%)	Emiratis	43	3.4
		Arabs & Iranians	346	27.4
	South Asians **(SA)**	Indians, Pakistanis & Bangladeshis	728	57.5
	Others (n=148)	South East Asians	70	5.5
		Africans	50	4.0
		European/ American/Australian)	28	2.2
	**Total**		**1265**	**100**
	Not reported		102	-
**Education Level**	Secondary education & Less		106	7.8
	College / University		1252	92.2
	**Total**		**1358**	**100**
	Not reported		9	-
**Economic status**	Inadequate		66	5.3
	Just adequate		480	38.3
	Well-to-do		625	49.9
	Extremely well -to-do		81	6.5
	**Total**		**1252**	**100**
	Not reported		115	-

**Table 2:** Knowledge of warning signs and presenting symptoms of Coronary heart disease (CHD)

Statements	Total Population N=1367				YAP Gender Distribution N=695			GP Gender Distribution N=662			YAP Distribution based on Ethnicity N=624				GP Distribution based on Ethnicity N=641			
	YAP N=701 (%)	GP N=666 (%)	Total N=1367 (%)	P Value	Male N=343 (%)	Female N=352 (%)	P Value	Male N=426 (%)	Female N=236 (%)	P Value	ME N=212 (%)	SA N=334 (%)	Others N=78 (%)	P Value	ME N=177 (%)	SA N=394 (%)	Others N=70 (%)	P Value
Definition of CHD	53.5	76.3	64.6	< 0.05	55.1	52.6	NS	76.3	76.7	NS	57.1	58.1	64.1	NS	74.0	78.9	70.0	NS
Warning signs:- Ordinary physical activity causing:																		
Excessive tiredness	42.1	46.4	44.2	< 0.05	37.6	46.3	< 0.05	43.0	52.5	< 0.05	47.6	38.3	41.0	NS	51.4	43.1	54.3	NS
Rapid heartbeat	60.3	49.1	54.9	<0.001	62.4	58.2	NS	46.7	53.4	NS	64.2	56.9	65.4	NS	54.8	44.7	64.3	< 0.05
Difficulty in breathing	66.9	62.5	64.9	< 0.05	65.9	68.2	NS	61.5	64.4	NS	61.3	68.9	73.1	NS	59.3	63.5	72.9	NS
Chest pain or discomfort	75.2	80.8	77.9	< 0.05	74.9	76.4	NS	80.0	82.2	NS	66.5	84.1	82.1	<0.001	68.9	86.8	82.9	<0.001
Presenting symptoms of heart attack																		
Pain or discomfort in the jaw	21.0	26.3	23.6	< 0.05	21.3	20.5	NS	26.3	26.3	NS	18.9	24.6	14.1	NS	18.1	30.5	28.6	< 0.05
Pain or discomfort in the neck or back	28.4	32.7	30.5	< 0.05	25.1	31.5	< 0.05	31.9	34.7	NS	25.0	25.7	26.9	NS	23.2	36.3	41.4	< 0.05
Pain or discomfort in the left shoulder	44.5	61.3	52.7	<0.001	36.2	52.8	<0.001	59.4	64.0	NS	42.5	47.0	35.9	NS	59.3	64.7	45.7	< 0.05
Pain or discomfort in the chest	76.7	83.0	79.8	<0.05	73.5	80.1	<0.05	82.6	83.9	NS	74.1	79.0	73.1	NS	79.1	86.5	77.1	< 0.05
Feeling nausea and gastric discomfort	28.7	28.7	28.7	NS	29.2	28.4	NS	26.5	33.1	< 0.05	31.1	25.1	35.9	NS	23.7	31.2	27.1	NS
Shortness of breath (difficulty in breathing)	66.3	65.8	66.1	NS	65.6	67.9	NS	63.1	69.9	< 0.05	68.4	61.4	71.8	NS	72.3	62.2	72.9	< 0.05
Breaking out in cold sweat	41.9	45.2	43.5	< 0.05	43.1	41.5	NS	42.0	50.4	< 0.05	41.5	45.5	42.3	NS	53.7	43.1	38.6	< 0.05
Feeling weak, lightheaded or faint	53.6	46.2	50.0	< 0.05	53.9	54.0	NS	44.8	48.3	NS	60.8	49.7	55.1	< 0.05	57.6	40.4	54.3	< 0.001
Overall satisfactory knowledge score > 6 out of 13 (Median score = 7)	52.8	56.8	54.7	< 0.05	50.7	55.7	< 0.05	53.3	62.7	< 0.05	52.4	54.2	61.5	NS	57.6	56.3	60.0	NS
GP = General population YAP = Young adult population ME = Middle Easterners SA = South Asians NS = Non-Significant																		

**Table 3:** Knowledge of risk factors for Coronary heart disease (CHD)

Statements	Total Population N=1367				YAP Gender Distribution N=695			GP Gender Distribution N=662			YAP Distribution based on Ethnicity N=624				GP Distribution based on Ethnicity N=641			
	YAP N=701 (%)	GP N=666 (%)	Total N=1367 (%)	P Value	Male N=343 (%)	Female N=352 (%)	P Value	Male N=426 (%)	Female N=236 (%)	P Value	ME N=212 (%)	SA N=334 (%)	Others N=78 (%)	P Value	ME N=177 (%)	SA N=394 (%)	Others N=70 (%)	P Value
Risk of CHD is high for women who smoke + use oral contraceptives	27.4	39.6	34.4	<0.001	26.8	28.1	NS	37.8	42.8	NS	30.7	26.0	29.5	NS	35.6	42.1	35.7	NS
Risk of CHD increases in women after menopause	19.4	24.6	22.6	< 0.05	16.9	22.2	NS	18.1	36.4	< 0.001	20.3	17.7	24.4	NS	31.1	21.3	24.3	< 0.05
Survivors of heart attack are at high risk of recurrences and at high risk of dying from them	60.4	64.8	62.6	< 0.05	58.9	58.5	NS	59.6	68.2	< 0.05	55.2	65.0	61.5	< 0.05	63.8	63.2	62.9	NS
Which of the following increases the risk of getting CHD:																		
Tobacco smoking	83.0	85.0	84.0	NS	86.3	80.4	< 0.05	83.1	89.0	< 0.05	86.3	84.4	82.1	NS	85.9	84.5	88.6	NS
High blood pressure	78.3	83.8	81.0	< 0.05	79.9	77.0	NS	82.4	86.9	NS	79.7	77.2	88.5	NS	79.7	85.0	85.7	NS
High blood cholesterol	77.7	84.7	81.1	< 0.05	76.1	79.5	NS	83.8	86.9	NS	76.4	82.3	70.5	< 0.05	82.5	85.8	84.3	NS
Physical inactivity	65.8	68.6	67.2	< 0.05	65.6	65.9	NS	68.5	68.6	NS	65.6	69.2	61.5	NS	71.2	66.8	80.0	NS
Obesity and overweight	76.9	80.6	78.7	< 0.05	75.5	78.4	NS	76.8	88.6	< 0.001	76.9	81.4	75.6	NS	83.6	80.5	84.3	NS
Diabetes mellitus	52.4	54.5	53.4	NS	51.6	52.6	NS	52.8	57.6	NS	51.9	52.7	50.0	NS	52.5	55.3	60.0	NS
Stress	68.3	77.9	73.0	<0.001	66.5	69.9	NS	75.8	81.4	NS	60.8	72.2	71.8	< 0.05	65.5	84.3	77.1	<0.001
Unhealthy diet	69.3	75.8	72.5	< 0.05	65.6	72.7	< 0.05	73.0	80.9	< 0.05	67.0	74.3	65.4	NS	72.9	78.4	80.0	NS
Family history	61.8	67.4	64.5	< 0.05	59.2	64.2	NS	63.6	74.2	< 0.05	59.9	65.0	57.7	NS	65.0	69.3	67.1	NS
Increasing age	50.4	44.7	47.6	NS	51.9	48.9	NS	43.4	47.0	NS	56.1	47.0	52.6	NS	54.2	38.8	60.0	<0.001
Being Male (CHD more common in men)	25.4	28.4	26.8	NS	27.7	23.0	NS	29.1	27.1	NS	26.9	26.0	19.2	NS	27.1	30.2	22.9	NS
Regular exposure to secondhand smoke increases the risk of heart disease by around 25%	49.5	56.0	52.7	<0.001	51.6	48.0	NS	55.9	56.4	NS	58.0	50.6	46.2	NS	61.6	53.8	57.1	NS
Overall satisfactory knowledge score ≥ 8 out of 15 (Median score = 9)	69.6	74.2	72.0	< 0.05	69.4	69.9	NS	70.0	78.4	< 0.05	71.7	73.4	67.9	NS	72.9	73.1	78.6	NS
GP = General population YAP = Young adult population ME = Middle Easterners SA = South Asians NS = Non-Significant																		
